# Distinct Stress Response and Altered Striatal Transcriptome in Alpha-Synuclein Overexpressing Mice

**DOI:** 10.3389/fnins.2018.01033

**Published:** 2019-01-10

**Authors:** Zinah Wassouf, Thomas Hentrich, Nicolas Casadei, Mirko Jaumann, Marlies Knipper, Olaf Riess, Julia M. Schulze-Hentrich

**Affiliations:** ^1^Institute of Medical Genetics and Applied Genomics, University of Tübingen, Tübingen, Germany; ^2^Molecular Physiology of Hearing, Department of Otolaryngology, Tübingen Hearing Research Centre, University of Tübingen, Tübingen, Germany

**Keywords:** alpha-synuclein, chronic unpredictable mild stress, Parkinson’s disease, transcriptome, psychiatric-related behavior

## Abstract

Parkinson’s disease (PD) is a progressive neurodegenerative disorder with motor symptoms and a plethora of non-motor and neuropsychiatric features that accompany the disease from prodromal to advanced stages. While several genetic defects have been identified in familial forms of PD, the predominance of cases are sporadic and result from a complex interplay of genetic and non-genetic factors. Clinical evidence, moreover, indicates a role of environmental stress in PD, supported by analogies between stress-induced pathological consequences and neuronal deterioration observed in PD. From this perspective, we set out to investigate the effects of chronic stress exposure in the context of PD by using a genetic mouse model that overexpresses human wildtype *SNCA*. Mimicking chronic stress was achieved by adapting a chronic unpredictable mild stress protocol (CUMS) comprising eight different stressors that were applied randomly over a period of eight weeks starting at an age of four months. A distinctive stress response with an impact on anxiety-related behavior was observed upon *SNCA* overexpression and CUMS exposure. *SNCA*-overexpressing mice showed prolonged elevation of cortisol metabolites during CUMS exposure, altered anxiety-related traits, and declined motor skills surfacing with advanced age. To relate our phenotypic observations to molecular events, we profiled the striatal and hippocampal transcriptome and used a 2 × 2 factorial design opposing genotype and environment to determine differentially expressed genes. Disturbed striatal gene expression and minor hippocampal gene expression changes were observed in *SNCA*-overexpressing mice at six months of age. Irrespective of the CUMS-exposure, genes attributed to the terms neuroinflammation, Parkinson’s signaling, and plasticity of synapses were altered in the striatum of *SNCA*-overexpressing mice.

## Introduction

Parkinson’s disease (PD) is a slowly progressive neurodegenerative movement disorder with age being its strongest risk factor (de Lau and Breteler, 2006; Emamzadeh and Surguchov, 2018). Besides *SNCA* and *LRRK2*, both responsible for autosomal dominant forms of PD (Lesage and Brice, 2009; Cookson, 2010), several causative mutations were identified together with susceptibility loci associated with disease risk (Nalls et al., 2014). In the *SNCA* gene, point mutations as well as duplication and triplication of that locus cause highly penetrant early onset PD with varying pathology and clinical features among patients (Polymeropoulos et al., 1997; Krüger et al., 1998; Singleton et al., 2003; Zarranz et al., 2004; Proukakis et al., 2013). Further, genetic variants in *SNCA* are associated with increased susceptibility for idiopathic PD (Satake et al., 2009; Simon-Sanchez et al., 2009). Yet, the heritable monogenic forms of PD are rare and account for less than 10% of all cases (Lesage and Brice, 2009) leaving the majority of cases without known etiology.

Following the first observation that exposure to 1-methyl-4-phenyl-1,2,3,6-tetrahydropyridine (MPTP) can trigger a parkinsonian phenotype (Ballard et al., 1985), numerous epidemiological studies were initiated to explore the contribution of environmental factors to the disease pathogenesis, and point towards a complex interplay between genetic and environmental factors (Ascherio and Schwarzschild, 2016). Exposure to environmental toxicants such as pesticides, solvents, and metals as well as traumatic brain injury are associated with higher risk for PD, while protective factors include tobacco, coffee, and physical activity (Ascherio and Schwarzschild, 2016). In addition, commonalities between stress-triggered pathological consequences and the neuronal atrophy observed in PD and other neurodegenerative diseases, suggest stress as an environmental risk factor associated with onset and progression of neurodegeneration (Smith et al., 2002). The initial observations of neuronal damage and synaptic dysfunction after stress exposure (Uno et al., 1989; Mizoguchi et al., 2000; Dias-Ferreira et al., 2009) are supported by clinical studies indicating a role for stress (early and chronic) and elevated glucocorticoids in age-related disorders like Alzheimer’s disease (AD), PD, and dementia (Borenstein et al., 2006; Simard et al., 2009; Rothman and Mattson, 2010; Zou et al., 2013). In accordance with these findings, experimental studies with toxin-induced models show that stress accelerates neurodegeneration and aggravates motor symptoms (Pienaar et al., 2008; Smith et al., 2008; Lauretti et al., 2016). Besides stress effects on motor symptoms of PD, evidence also points to an altered stress response and consequences for non-motor and neuropsychiatric symptoms partially characterizing the prodromal disease phase (Savica et al., 2010; Goldman and Postuma, 2014). Such effects are believed to elicit from modulation of the dopaminergic system including the limbic circuitry upon stress exposure.

Most experimental studies investigating the effect of stress in PD, including the above mentioned, used toxin-induced models, in which dopamine depletion, mitochondrial dysfunction, or ROS production resulting in neurodegeneration are rapidly and irreversibly induced. Here, we use a pre-symptomatic genetic PD mouse model, overexpressing human *SNCA* (Yamakado et al., 2012), with motor deficits that develop slowly with increasing age, to reveal effects of chronic unpredictable mild stress on motor and non-motor features. We provide evidence on altered stress response and anxiety-associated behavior in *SNCA*-overexpressing mice preceding the occurrence of motor deficits. Combining these data with striatal transcriptome profiling pointed at accompanied disturbances in genes and pathways associated with *SNCA* pathology.

## Materials and Methods

### Animals, Study Design, and the Chronic Unpredictable Mild Stress (CUMS) Protocol

The transgenic mouse model was generated using a *Bacterial Artificial Chromosome* (BAC) construct comprising a fused PAC AF163864 and BAC AC097478 which contains the entire human *SNCA* gene locus with 28 kb 5′- and 50 kb 3′-flanking regions (Yamakado et al., 2012). Animals were bred on a C57BL/6N background as described previously (Nuber et al., 2013). Using PCR, genotyping was done with DNA isolated from ear biopsies before weaning. Homozygosity was confirmed with quantitative real-time PCR performed on a *LightCycler 480* (Roche). Only homozygous mice were used for this study.

At the age of 14 weeks, wildtype (WT) and *SNCA*-overexpressing (TG) female mice were randomly assigned to either the standard (SE) or the CUMS (ST) conditions, which resulted in four experimental groups, WT_SE_, WT_ST_, TG_SE_, and TG_ST_, that at the age of six months underwent either behavioral tests or transcriptome profiling.

An additional group of male WT and TG mice was housed in a standard environment and used for motor behavioral characterizations at 13 and 20 months of age.

**FIGURE 1:**
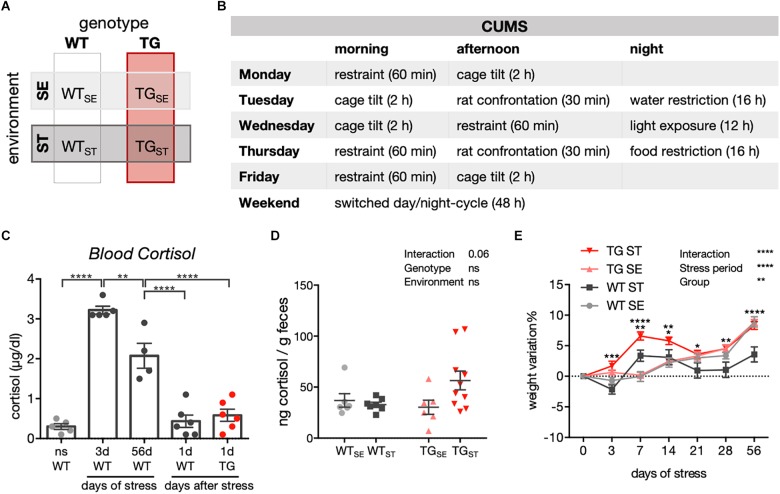
CUMS-induced effects on cortisol levels and body weight. **(A)** Schematic diagram showing experimental design and groups of WT and TG mice allocated to either standard (SE) or stress-inducing (ST) environments. **(B)** Chronic unpredictable mild stress protocol for one week, repeated over eight weeks. **(C)** Blood cortisol measurement in WT and TG mice exposed to CUMS. ns, not stressed; 3d: 3 days of CUMS; 56d: 56 days of CUMS; 1d: WT and TG one day after CUMS. One-way ANOVA was performed followed by Tukey’s correction for multiple comparisons (*n* = 4–6). Data presented as individual points with mean ± SEM. **(D)** Cortisol measurement in feces of WT and TG mice in SE and during the CUMS protocol (ST). Data presented for individual mice together with mean ± SEM for each group (*n* = 6–10). **(E)** Weight variation in WT and TG mice during CUMS (*n* = 23–25). Repeated measures two-way ANOVA was performed followed by Tukey’s correction for multiple comparisons. Data presented as mean ± SEM. ^∗∗∗^WT_ST_ vs. TG_ST_ (at 3 days). ^∗∗^WT_SE_ vs. WT_ST_ and WT_ST_ vs. TG_ST_, ^∗∗∗∗^TG_SE_ vs. TG_ST_ (at 7 days). ^∗^WT_ST_ vs. TG_ST_, ^∗∗^TG_SE_ vs. TG_ST_ (at 14 days). ^∗^WT_ST_ vs. TG_ST_ (at 21 days). ^∗∗^WT_ST_ vs. TG_ST_ (at 28 days). ^∗∗∗∗^WT_SE_ vs. WT_ST_ and WT_ST_ vs. TG_ST_ (at 56 days).

The CUMS paradigm was adapted from previously described protocols (Li et al., 2015). The chronic stress lasted for eight weeks and included eight different stressors, which were applied randomly. These stressors comprised the following: restraint stress, cage tilt, rat confrontation, food restriction, water restriction, extended light exposure (36 h), and reversed light/dark cycle (Figure 1B). In SE, groups of three to four mice were housed in standard cages (365 × 207 × 140 mm, *Type II long*) with normal light/dark cycle (12 h light /12 h dark) and free access to food and water.

### Ethics Statement

All procedures strictly adhered to international standards for the care and use of laboratory animals and were approved by the local Animal Welfare and Ethics committee of the Country Commission Tübingen, Germany (TVA HG 4/12).

### Blood Cortisol Measurement

For blood cortisol measurements, mice were sacrificed by cervical dislocation followed by decapitation within minutes from disturbing the home cage. Trunk blood was collected, incubated at room temperature for 2 h then centrifuged at 1,000 × *g* for 10 min before serum was collected and stored frozen until measurement.

Blood cortisol level in mice after three and 56 days of stress was measured after applying movement restraint stress. Mice were then sacrificed by cervical dislocation and decapitation and serum was collected as mentioned above. Mice from all groups were sacrificed between 11:30 am and 12:30 pm. Cortisol measurements were performed by IDEXX BioResearch, Ludwigsburg, Germany.

### Measurement of Fecal Cortisol Metabolites

Fecal pellets were collected from mice between two stress sessions during CUMS. To extract steroids from feces, *DetectX*^TM^ *Steroid Solid Extraction* kit was used following the manufacturer’s protocol. In short, 0.2 g of fecal solid and 2 ml ethanol were mixed vigorously for at least 30 min. Samples were centrifuged at 5,000 rpm for 15 min, then 1.5 ml of supernatant was transferred to a clean tube and evaporated to dryness in a SpeedVac centrifuge. Extracted samples were reconstituted with ethanol and buffer to a final ethanol concentration of 5%.

Cortisol was measured using the *DetectX*^TM^ *Cortisol Enzyme Immunoassay* kit following the manufacturer’s protocol. In short, 50 μl of samples were added into wells of an assay plate. 25 μl of DetectX^TM^ Cortisol Conjugate was added followed by 25 μl of the DetectX^TM^ Cortisol Antibody. The plate was sealed and gently shaken for 1 h at RT. Each well was aspirated and washed four times with 300 μl wash buffer. 100 μl of TMB substrate was added and incubated 30 min at RT without shaking. Optical density was measured using BMG FLUOstar OPTIMA Microplate Reader. The results were analyzed using the MyAssay (MyAssays Ltd.) software.

### Animal Behavior

Behavioral experiments were performed with the experimental groups; WT_SE_, WT_ST_, TG_SE_, and TG_ST_ at six months of age. In addition, groups of male WT and TG mice were used for motor behavioral experiments at 13 and 20 months of age. As some animals died, sample size varied between the 13- and 20-month time points. Mice were allowed to acclimate to the experimental room 30 min before testing and all experiments were performed during the dark phase by an experimenter blind to the mice’s genotype and group. Mice’s weight was monitored before and throughout the CUMS period.

#### Light/Dark Box

Anxiety-related behavior in mice was tested with a light/dark box apparatus consists of a dark (one-third) and an illuminated (light) compartment (two-third) with an opening of 10 × 10 cm between both. Mice were placed into the illuminated compartment (∼420 lux) facing the opening to the dark compartment and allowed to freely explore both areas for a total of 10 min. Mice movement was videotaped and tracked using TSE VideoMot 2 equipment (TSE systems). Time spent and distance traversed in light were analyzed and plotted for each animal.

#### Elevated Plus Maze

Mice exploratory behavior on an elevated plus maze was assessed at six months of age. The setup comprised two open and two closed arms elevated 50 cm above ground and illuminated with soft white light (∼100 lux). Mice were placed in the intersection of the four arms facing an open arm opposite to the experimenter and allowed to freely explore the maze for a total of 10 min. Mice movement was videotaped and tracked using TSE VideoMot 2 (TSE systems). Visits, time spent, and distance traversed on open arms were assessed and presented for each animal.

#### LabMaster

An automated home cage system equipped with infrared light beams (*LabMaster*, TSE systems) was used to detect animals’ locomotion. The total activity and movement in center and periphery were recorded, together with drinking and feeding behavior, in 1-min sample interval over a total period of 22 h of recording. WT_SE_, WT_ST_, TG_SE_, and TG_ST_ mice groups were tested before and after CUMS exposure, in addition to WT and TG mice at 13 and 20 months of age. The recording started 5 min before the onset of the dark phase. Animals’ weight was monitored before and after the test period.

#### CatWalk

In order to assess motor performance, a detailed gait analysis using a *CatWalk* system (*CatWalk XT 10.0*; Noldus) was performed for mice from WT_SE_, WT_ST_, TG_SE_, and TG_ST_ groups, in addition to 13- and 20-month-old WT and TG mice. Mice were allowed to cross a walkway composed of an illuminated glass plate and a high-speed camera positioned underneath. When paws touch the glass plate, light gets scattered and captured by the camera. Patterns linked to paws, stride, and gait were derived and analyzed. Only animals with at least one straight track without any interruption were included in the analysis.

#### Challenging Beam Walking

Animals were assessed with respect to their sensorimotor function on a challenging beam composed of four bars (25 cm each) of different width (3.5–0.5 cm). Starting at the wider bar, mice were trained to traverse across the bars of smaller width toward a home cage placed at the end of the beam. After 2 days of training (three trials per day for each animal), a mesh grid was placed on top of the beam and the mice’s movements were video recorded over five trials for each animal. Paw slips were counted for each trial.

### Tissue Preparation for RNA Isolation

Mice for RNA isolation were randomly chosen from each group and sacrificed with cervical dislocation followed by head decapitation within 2 min from disturbing the home cage. Brains were removed and immediately dissected on ice, then snap frozen in liquid nitrogen.

### RNA Sequencing

The polyadenylated fraction of RNA isolated from striatal and hippocampal tissue (*n* = 3–4 animals in each of the four experimental groups) was used for single-end RNA-seq. Total RNA and DNA were simultaneously extracted using the *AllPrep DNA/RNA Mini Kit* (Qiagen). Quality was assessed with an *Agilent 2100 Bioanalyzer*. Samples with high RNA integrity number (RIN > 8) were selected for library construction. Using the *TruSeq RNA Sample Prep Kit* (Illumina) and 500 ng of total RNA for each sequencing library, poly(A) selected single-end sequencing libraries were generated according to the manufacturer’s instructions. All libraries were sequenced on an Illumina HiSeq 2500 platform at a depth of 10–20 million reads each. Library preparation and sequencing procedures were performed by the same individual and a design aimed to minimize technical batch effects was chosen.

### Bioinformatics

#### Quality Control, Alignment, and Expression Analysis

Read quality of RNA-seq data in fastq files was assessed using FastQC (v0.11.4) (Andrews, 2010) to identify sequencing cycles with low average quality, adaptor contamination, or repetitive sequences from PCR amplification. Reads were aligned using STAR (v2.4.2a) (Dobin et al., 2013) allowing gapped alignments to account for splicing against a custom-built genome composed of the Ensembl Mus musculus genome v82 and the human *SNCA* transgene. Alignment quality was analyzed using samtools (v1.1) (Li et al., 2009) and visually inspected in the Integrative Genome Viewer (v2.3.67) (Thorvaldsdottir et al., 2013). Normalized read counts for all genes were obtained using DESeq2 (v1.8.2) (Love et al., 2014). Transcripts covered with less than 50 reads were excluded from the analysis leaving 12,287 genes for determining differential expression in each of the pair-wise comparisons between experimental groups.

The 2 × 2 factorial design of the experiment was captured in a general linearized model in DESeq2 modeling expression (t) as a function of genotype (g), the environment (e), and their interaction (g × e). Surrogate variable analysis (sva, v3.22.0) was used to minimize unwanted variation between samples (Leek et al., 2012). Given that differences in transcript abundances in brain tissue are often small in magnitude and *in vivo* RNA-seq data are deemed to be more variable (Maze et al., 2014), we set | log_2_ fold-change|≥ 0.3 and adjusted *p*-value ≤ 0.15 to determine differentially expressed genes, as computationally predicted candidates down to the lower end of these thresholds could be confirmed in qPCR assays.

Gene-level abundances were derived from DESeq2 as normalized read counts and used for calculating the log_2_-transformed expression changes underlying the expression heatmaps with ratios computed relative to the mean expression in WT_SE_. The sizeFactor-normalized counts provided by DESeq2 also went into calculating nRPKMs (normalized Reads Per Kilobase per Million total reads) as a measure of relative gene expression as motivated before (Srinivasan et al., 2016). The sizeFactors further served in scaling estimated abundances derived from Salmon (v0.7.2) (Patro et al., 2016) when determining the transcript-level composition for individual genes.

#### Gene Annotation and Functional Analyses

All Gene ID conversions were done using biomaRt Bioconductor package (v2.32.1) querying v87 of the Ensembl database.

Canonical pathways between sets of DEGs as well as predicted relationships between DEGs and disease aspects/biofunctions were derived from Ingenuity Pathway Analysis (IPA, v01-12, Qiagen).

### Reverse Transcription-Quantitative PCR (RT-qPCR)

RT-qPCR was performed to validate RNA-seq results for *Slc6a11*, *Slc5a7*, and *Aldh1a1* genes. 100 ng of total RNA was used for the reverse transcription reaction (*QuantiTect Reverse Transcription* kit, Qiagen) following the manufacturer’s instructions. The resulting cDNA was diluted (1:20) and 2 μl were used for the qPCR assay, mixed with primers (0.5 μM) and *SYBR green* master mix (Qiagen). Relative expression was calculated based on Pfaffl model (Pfaffl, 2001) after normalization to relative expression of the reference gene *Pgk1*, which was previously assessed for its stable expression using *BestKeeper* (Pfaffl et al., 2004), *Normfinder* (Andersen et al., 2004), and *Genorm* (Vandesompele et al., 2002). Data were calculated using Excel-based equations and further validated using *qBase* (Hellemans et al., 2007).

### Statistical Analysis

Statistical comparisons were done in GraphPad Prism (v6.0). Two-way ANOVA was used to test for genotype, environment or age, and their interaction. For weight variation during CUMS, repeated measures two-way ANOVA was performed with time (stress period) as a within-group factor and group (genotype and environment) as a between-group factor. The α-level was set to 0.05. Data presented as individual points together with mean ± SEM for each group. Ns, not significant, ^∗^*p* < 0.05, ^∗∗^*p* < 0.01, ^∗∗∗^*p* < 0.001, and ^∗∗∗∗^*p* < 0.0001.

Some behavioral data were excluded from analysis due to technical problems in data acquisition or based on predefined criteria (as explained below). The excluded values were spread equally among groups.

In light/dark box: one animal was excluded from the analysis due to tracking software errors. Elevated plus maze: one outlier was identified based on ROUT method (Motulsky and Brown, 2006) and removed from WT_SE_ group. LabMaster: one animal was excluded from water intake measurements due to water leaking during test, one animal was excluded from post-stress activity measurements due to tail injury, one animal was excluded from pre-stress activity measurements due to errors in data recording, and one outlier was identified by ROUT method and removed from pre- and post-stress analyses. In CatWalk assay: only animals with at least one straight track without any interruption were included in the analysis. Challenging beam walking: one animal from TG_20m_ group refused to cross the beam during test. Data presented for individual mice and number of animals analyzed are indicated for each test in the figure legends. Results from two-way ANOVAs for the comparisons WT_SE_ vs. WT_ST_, WT_SE_ vs. TG_SE_, WT_ST_ vs. TG_ST_ and TG_SE_ vs. TG_ST_ are presented.

## Results

### Chronic Mild Stress Exposure Elevated Cortisol Levels and Altered Weight Development in Wildtype and *SNCA*-Overexpressing Mice

To better understand effects of chronic stress on behavioral phenotypes and molecular disturbances in the context of PD, a transgenic mouse model overexpressing full-length human *SNCA* (TG) (Yamakado et al., 2012) together with wildtype controls (WT) were exposed to a chronic unpredictable mild stress (CUMS) protocol. Starting at 14 weeks of age, CUMS was applied over eight weeks, and compared to WT and TG mice that remained undisturbed in standard environment. Consequently, two genotypes (WT, TG) and two environmental conditions (SE, ST) were evaluated throughout the study (Supplementary Figure 1 and Figures 1A,B). CUMS resulted in elevated blood cortisol levels after 3 and 56 days of stress when measured directly after applying movement restraint stress. WT as well as TG mice showed cortisol levels similar to unstressed mice one day after eight weeks of stress exposure (Figure 1C), indicating an acute but no chronic rise in blood cortisol levels after CUMS (*n* = 4–6). In order to assess the acute response to stress from both WT and TG mice in the same behavioral cohort and to avoid the rapid stimulation of the endocrine system due to blood sampling and mice fixation, we measured fecal cortisol metabolites in samples collected in between two stress sessions during the CUMS course (*n* = 6–10). Here, a clear tendency for a prolonged increase of fecal cortisol metabolites was observed in feces from TG_ST_ compared to WT_ST_ and TG_SE_ groups (Figure 1D) pointing towards a difference between both genotypes in their immediate response to stress. In addition, whereas body weight developed similarly in WT and TG in standard environment (Figure 1E), CUMS led to differential weight development in WT_ST_ and TG_ST_ starting at 3 days of stress exposure and throughout the stress period (*n* = 23–25). This difference was not based on altered food or water intake as indicated in LabMaster system measurements (Supplementary Figure 2).

### *SNCA* Overexpression Influenced Anxiety-Like Behavior

**FIGURE 2:**
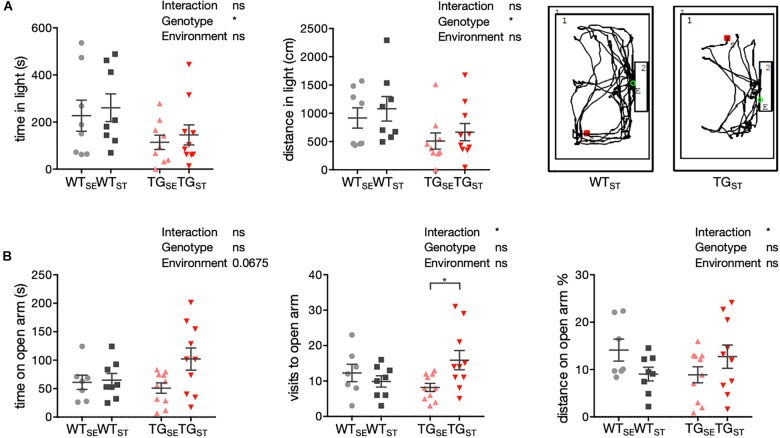
Anxiety-related phenotypes with genotype-specific CUMS effects. **(A)** To assess anxiety-related behavior in WT and TG mice (*n* = 8–10) a light/dark box test was used. Time in seconds (s) and distance (cm) in light are plotted for individual mice together with mean ± SEM for each group. Right panel: Representative tracks for WT_ST_ and TG_ST_ in the lit box. **(B)** Anxiety-related behavior in the EPM test. Time (s), visits, and distance (% from total distance on the maze) on open arms are shown for individual WT and TG mice (*n* = 7–10) with mean ± SEM for each group. Two-way ANOVA followed by Tukey’s correction for multiple comparisons was performed.

To assess anxiety-related phenotypes in TG compared to WT mice and test the genotype-dependent influence of CUMS, behavior in a light/dark box and elevated plus maze was studied. In the light/dark box, mice were exposed to a novel environment with brightly illuminated and dark “safe” areas, in which innate aversion and novel spontaneous exploratory mice behavior were observed and recorded (*n* = 8–10). Tracking distance crossed and time spent in the lit compartment, an increase in anxiety-like behavior was evident for TG mice irrespective of the environmental condition (Figure 2A). Comparing the experimental groups with two-way ANOVA showed a significant genotype effect, but no significant environment effect or interaction of both.

In the elevated plus maze, mice were confronted with a novel environment consisting of two elevated open and two closed arms. This set-up generates fear of height and open areas opposing the mice’s spontaneous exploratory behavior, used to evaluate the mice’s proclivity toward closed arms versus their explorative time spent on open arms (*n* = 7–10). In contrast to WT mice, TG mice exposed to CUMS spent more time and traveled greater distances on the open arms, reflected in a significant interaction of environment and genotype (Figure 2B).

### CUMS Led to Hypolocomotion in Both Wildtype and *SNCA*-Overexpressing Mice

**FIGURE 3:**
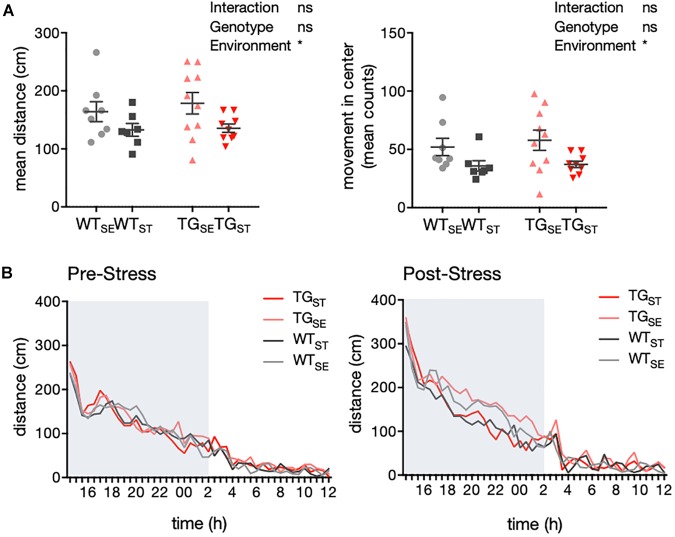
CUMS induced hypolocomotion in both WT and TG mice. **(A)** Activity of mice recorded in an automated homecage system (LabMaster) (*n* = 7–10). Mean distance crossed (cm) and movement in center during the dark phase are plotted for individual mice together with mean ± SEM for each group. **(B)** Circadian activity before and after CUMS. Plotted is mean distance crossed (cm) every 30 min over 22 h total recording time (pre-stress *n* = 11–12, post-stress *n* = 7–10). Shading indicates the dark phase.

In order to explore the baseline locomotor behavior, we used a homecage-like environment that minimizes the influence of unfamiliar surroundings and requires minimal animal handling prior to the test. Measurements for activity as well as drinking and feeding behavior were carried out with an automated homecage system (LabMaster, TSE) in WT and TG mice before and after CUMS exposure. During the dark phase of the observation period, CUMS-exposed WT and TG mice showed less traveling distance and movement in the center of the cage compared to animals housed in standard environment (*n* = 7–10) (Figure 3A). Hence, the effect on locomotor activity was specific to CUMS exposure and similar in both genotypes, supported by the fact that the circadian activity measurements prior to CUMS showed similar rhythms in all four groups (pre-stress *n* = 11–12, post-stress *n* = 7–10) (Figure 3B).

### Impaired Motor Performance Surfaced With Advanced Age in *SNCA*-Overexpressing Mice

**FIGURE 4:**
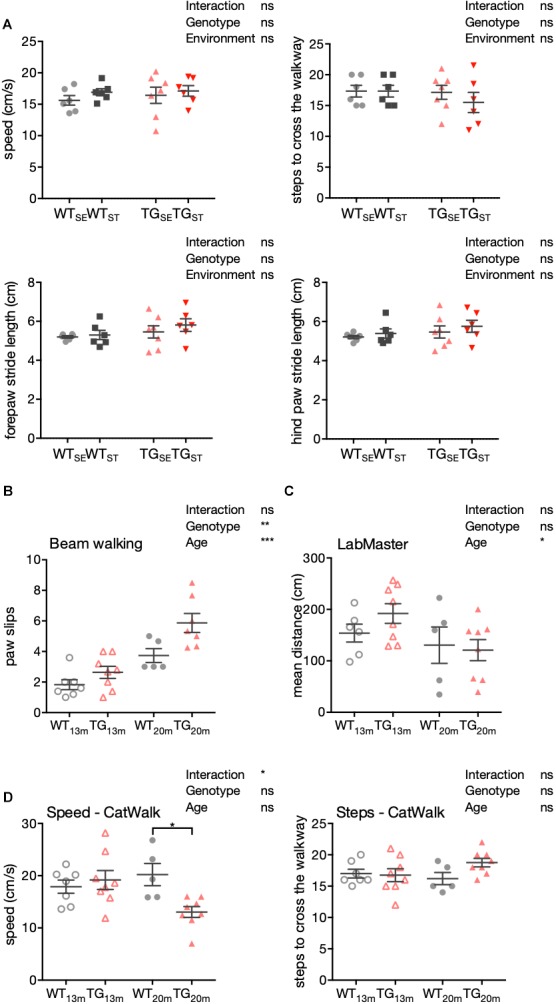
Impaired motor performance in 20-month-old TG mice. **(A)** Detailed gait analysis using the CatWalk system. Speed in cm/s, number of steps to cross the walkway, forepaws and hind paws stride length are plotted as individual data points with mean ± SEM for each group. **(B)** Mean number of paw slips in a challenging beam walking test is shown with 13- and 20-month-old WT and TG mice. Plotted are data points for individual mice with mean ± SEM for each group. **(C)** Mean distance crossed (cm) in LabMaster is shown with 13- and 20-month-old WT and TG mice. **(D)** Speed (cm/s) and steps in CatWalk system are shown with 13- and 20-month-old WT and TG mice. Plotted are data points for individual mice with mean ± SEM for each group. Two-way ANOVA with Bonferroni’s correction for multiple comparisons was performed (*n* = 5–8).

To further extend our behavioral characterization to aspects of motor performance, we performed a detailed gait analysis using a CatWalk system (CatWalk XT 10.0; Noldus). Parameters capturing speed, steps to cross the walkway, fore- and hind paws stride length showed no significant differences between the experimental groups at an age of six months (Figure 4A). Accordingly, these results indicate that the observed increase in anxiety-like behavior in TG mice (Figure 2A), occurred without obvious motor impairment or changes in gait.

In keeping with these results, signs for declined motor abilities were first observed in 13- and 20-month-old male *SNCA*-overexpressing mice with respect to their sensorimotor functions, general locomotion, and gait. Compared to WT, TG mice showed an increased number of paw slips in the challenging beam walk test with an additional significant main effect in age (Figure 4B). While in LabMaster, the mean distance was affected by age similarly in both genotypes (Figure 4C), CatWalk experiments showed slower movement and tendency to more steps in TG mice with increasing age (*n* = 5–8) (Figure 4D).

### *SNCA* Overexpression Disturbed the Striatal Transcriptome Largely Irrespective of CUMS-Exposure

**FIGURE 5:**
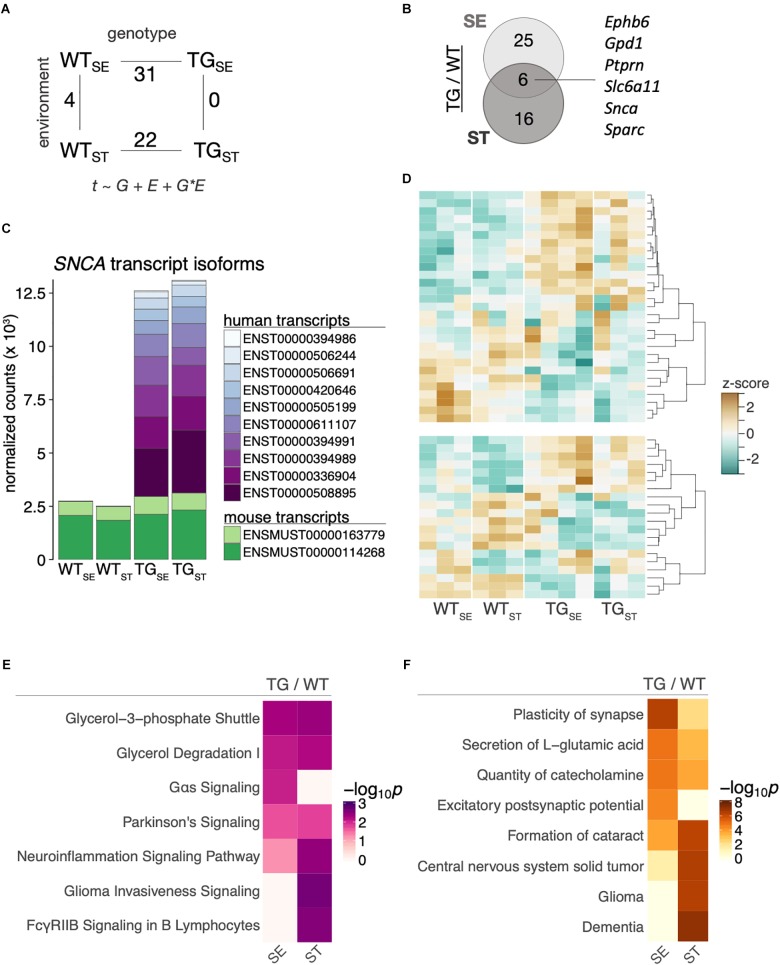
*SNCA* overexpression altered the striatal transcriptome in TG mice. **(A)** Number of differentially expressed genes for the main comparisons between the four experimental groups in a 2 × 2 factorial design. Gene expression (t) was modeled as a function of genotype (G), environment (E), and their interaction (G × E). **(B)** Venn diagram showing overlap of differentially expressed genes between TG and WT mice in the tested environmental conditions. **(C)** Composition and expression level of striatal murine and human *SNCA* splice variants. **(D)** Heatmap of hierarchically clustered *z*-scores across all experimental groups for differentially expressed genes that were identified in comparing TG_SE_/WT_SE_ (upper panel) and TG_ST_/WT_ST_ (lower panel). High expression is marked by the orange color spectrum, low expression by turquoise colors. **(E)** Canonical pathway analysis identified significantly enriched cellular pathways among DEGs derived in the tested environmental conditions. Significance values color-coded and hierarchically clustered representing likeliness of pathway participation. **(F)** Disease aspects and biological functions predicted to be affected based on DEGs derived in the tested environmental conditions. Significance values color-coded and hierarchically clustered representing likeliness of downstream effects on disease aspects.

To relate the observed behavioral changes upon CUMS-exposure in the context of *SNCA* overexpression to alterations on a molecular level, we profiled the striatal and hippocampal transcriptome of 6-month-old mice from the four experimental groups using RNA-seq. Differentially expressed genes were determined in a 2 × 2 factorial design with gene expression (t) modeled as a function of genotype (G), environment (E), and their interaction (G × E). Comparing *SNCA*-overexpressing mice to their wildtype controls, 31 and 22 genes were differentially expressed (DEGs) in the striatum under standard environment and following stress exposure respectively, (Figure 5A) with six common DEGs in both comparisons (Figure 5B). Among them was the transgene itself, for which we explored the changes in striatal *SNCA* transcripts by aligning against a custom-built genome composed of the *Mus musculus* genome and the human *SNCA* transgene. This approach revealed similar endogenous transcript levels in all groups and an addition of human *SNCA* splice variants in TG mice that caused ∼5-fold overexpression in both environmental conditions (Figure 5C).

While the relatively small overlap of only six genes in Figure 5B suggested a stress-dependent modulation of the *SNCA*-induced disturbances, most genes showed similar trends of expression changes across both environmental conditions (Figure 5D). In line, CUMS exposure led to no significant gene expression changes in TG animals and four DEGs in WT animals (Figure 5A and Supplementary Figure 3). To further explore *SNCA*-induced disturbances, we used pathway-analysis tools to comparatively visualize enriched cellular pathways (Figure 5E) as well as disease aspects and biological functions (Figure 5F) for standard environment and CUMS-exposure. In line with Gene Ontology terms previously linked to *SNCA* overexpression (Borrageiro et al., 2018; Wassouf et al., 2018), terms like *Neuroinflammation Signaling Pathway* and *Plasticity of synapse* were among the enriched pathways and cellular functions.

**FIGURE 6:**
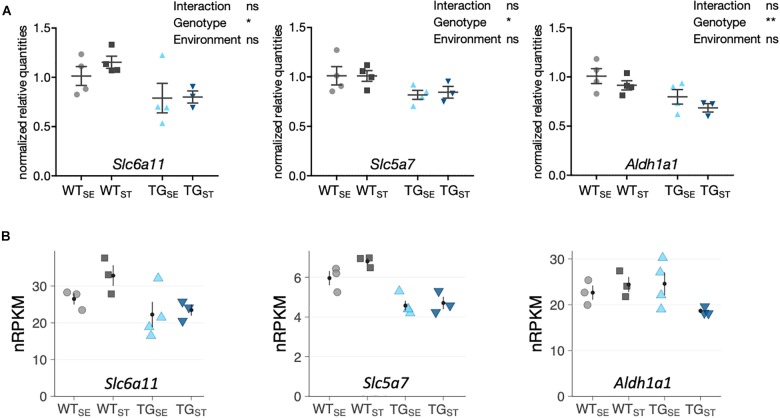
qPCR validation of selected genes altered by *SNCA* overexpression. **(A)** Reverse transcription-quantitative PCR results for selected genes shown as normalized quantities relative to WT_SE_ (*n* = 3– 4) and plotted as individual data points with mean ± SEM. Two-way ANOVA was performed. **(B)** RNA-seq results for the same genes. Shown are expression levels in normalized reads per kilobase per million (nRPKMs) as individual data points with mean ± SEM.

To validate that most *SNCA*-induced disturbances were not affected by CUMS-exposure, gene expression changes were assessed for selected candidates using quantitative PCR. Agreeing with RNA-seq results, *Slc6a11* (Solute Carrier Family 6 Member 11)*, Slc5a7* (Solute Carrier Family 5 Member 7) *and Aldh1a1* (Aldehyde Dehydrogenase 1 Family Member A1) showed similar disturbances in TG mice in both tested environments (Figure 6).

In the hippocampus, compared to previously described disturbances at 12 months of age (Wassouf et al., 2018) only few genes were differentially expressed in 6-month-old TG mice (Supplementary Figure 4). While not significant, expression of the glucocorticoid receptor (GR) showed a trend of downregulation after CUMS exposure and in TG mice (Supplementary Figure 4), indicating a modest effect of the CUMS paradigm on gene expression in the context of *SNCA* overexpression.

## Discussion

Taken together, using a genetic mouse model overexpressing human *SNCA* under its native promotor, we describe effects of chronic unpredictable mild stress on PD motor and non-motor features by combining phenotypic observations with molecular insights from striatal and hippocampal gene expression data sets. We observed specific stress responses upon *SNCA* overexpression with an impact on neuropsychiatric-related behavior, while motor abilities remained unchanged. These observations were accompanied by disturbed striatal gene expression in *SNCA*-overexpressing mice, with only minor changes on gene expression in the hippocampus at 6 months of age (Supplementary Figure 4).

Specifically, using a light/dark box test, *SNCA*-overexpressing mice showed increased anxiety-associated symptoms irrespective of the environmental condition, while the elevated plus maze gave discordant results and higher tendencies to explore the open arms for TG mice exposed to CUMS. These observations were associated with prolonged higher cortisol metabolites in feces from TG mice when samples in between two consecutive stressors in the CUMS protocol were taken. Owing to the fact that blood samples showed no chronic rise in cortisol levels in either WT or TG mice after one day following the CUMS protocol, we propose *SNCA*-overexpressing mice to have a prolonged response to acute stressors when compared to their WT counterparts. Of note, no baseline levels were measured prior to CUMS in these mice and only matched controls (WT littermates of same age, sex, and housing conditions) were used.

Anxiety-associated symptoms were assessed with the light/dark box and elevated plus maze tests, each of them using different conditions to evaluate mice aversions and spontaneous exploratory behavior. While the light/dark box utilizes mice aversion to a brightly lit area (Bourin and Hascoet, 2003), the elevated plus maze uses height and open areas to test mice exploration versus proclivity toward closed spaces (Walf and Frye, 2007). Given that the aversiveness of the test conditions is an important factor influencing the results, e.g., light condition, and due to the use of bright light (∼420 lux) in the light/dark box and softer light condition (∼100 lux) in the elevated plus maze, we suggest these factors to contribute to the observed discordance in both anxiety tests. Furthermore, the test condition in the light/dark box (e.g., the bright light) might pose a possible external stressor with an anxiogenic effect in both TG groups, presumably due to the protracted elevation of cortisol in TG mice following a stressful experience as suggested by the fecal cortisol metabolites measurement. Similarly, unexpected results after CUMS were reported before (Kompagne et al., 2008) and interpreted as a result of possible loss of interest in the environment and altered emotional processing. In addition, the observed anxiety-related behavior was accompanied by hypolocomotion in response to CUMS from both WT and TG, but no change in motor performance or gait was observed in TG mice. Consequently, declined motor skills are expected with increasing age in this model, as confirmed at an age of 13 and 20 months for a cohort of male mice.

In line with these results, a dysregulation of the hypothalamic–pituitary–adrenal (HPA) system and consequent sustained higher cortisol levels have been frequently described in patients with Alzheimer’s and Parkinson’s disease compared to healthy controls (Hartmann et al., 1997; Charlett et al., 1998). These clinical reports together with animal studies (Yau and Seckl, 1992; Mizobuchi et al., 1993) propose this dysregulation to elicit from a disturbed negative feedback response and reduced expression of hippocampal mineralocorticoid receptors (MR). However, hippocampal transcriptome data from the same cohort used in this study showed no significant change in MR or GRs. From this perspective, some non-motor features of PD can be seen as concomitants of the altered stress response, namely, anxiety and depression. With occurrence of almost 30%, anxiety and depression are relatively common features of the PD prodromal phase, where dopaminergic and non-dopaminergic neuronal circuitries are implicated (Goldman and Postuma, 2014; Bellou et al., 2016). Further, associated with anxiety, there are some PD characteristic traits such as lower novelty seeking and higher harm avoidance, both related to poor disease prognosis and suggested to result from damage to the mesolimbic dopaminergic projections involved in reward and motivation behavioral (Menza et al., 1993; Kaasinen et al., 2001; Costa and Caltagirone, 2015).

Considering reports on the aftermath of chronic stress on the brain describing several genetic and non-genetic interacting mechanisms (McEwen et al., 2015), we sought to link phenotypic observations to molecular events and biological pathways that are affected upon CUMS-exposure in the context of *SNCA* overexpression. Profiling the striatal transcriptome we observed similar gene expression changes in TG mice for both environmental conditions, indicating a dominant effect of *SNCA* overexpression on striatal gene expression. In line with previous reports on disturbed gene expression in brains of PD patients and mouse models (Borrageiro et al., 2018; Wassouf et al., 2018), altered genes were linked to synaptic function and neurotransmission. Using qPCR, the *SNCA*-induced disturbances observed by RNA-seq were confirmed for selected genes such as *Slc6a11*, *Slc5a7*, and *Aldh1a1*. While *Slc6a11* and *Slc5a7* are membrane-bound transporters related to GABA and cholinergic signaling pathways, respectively, *Aldh1a1* is involved in alcohol metabolism and other environment-induced metabolic responses, with an implication in PD and AD pathophysiology (Wey et al., 2012; Grunblatt and Riederer, 2016).

Both RNA-seq and qPCR results suggest a modest impact of chronic stress on these *SNCA*-induced gene expression changes. Importantly, inducing a stress response in rodents is highly dependent on the protocol used. Variables such as diversity and predictability, duration, and degree of stressors applied are affecting the type of response observed (Anisman et al., 1998; Razzoli et al., 2011; Savignac et al., 2011). In addition, different strains exhibit variations in stress susceptibility/resistance responses in mice, especially with C57BL/6 being less vulnerable to stress than other strains (Anisman et al., 1998; Razzoli et al., 2011; Savignac et al., 2011). Altogether, these considerations, supported by the absence of chronic elevation of blood cortisol in our cohorts, may explain the limited impact of CUMS-exposure on the striatal and hippocampal transcriptome.

In summary, this study provides evidence that *SNCA* overexpression modulates the stress response upon CUMS-exposure influencing psychiatric-associated behavior that precedes the manifestation of motor impairment in a genetic mouse model. On transcriptome level, genes attributed to neuroinflammation, Parkinson’s signaling, and plasticity of synapses were altered in the striatum of TG mice irrespective of the CUMS-exposure. Complementing these data with further physiological readouts and details on protein abundancies is pivotal to understand the complex gene-environment interplay in PD toward more personalized and effective treatments.

## Data Availability Statement

The datasets generated for this study can be found in GEO database, accession numbers GSE116009 and GSE116010.

## Author Contributions

JS-H initiated the study and designed the experiments with OR. ZW performed the experiments. NC guided the behavioral characterizations of the mouse model. MJ performed the cortisol metabolites measurement. TH and JS-H were responsible for computational analyses of the data. ZW, TH, and JS-H wrote the manuscript. All authors read and approved the manuscript.

## Conflict of Interest Statement

MJ is currently employed by HB Technologies AG. The remaining authors declare that the research was conducted in the absence of any commercial or financial relationships that could be construed as a potential conflict of interest.
