# Supplementary material for: Distinct Stress Response and Altered Striatal Transcriptome in Alpha-Synuclein Overexpressing Mice
Source: Front Neurosci. 2019 Jan 10;12:1033. doi: 10.3389/fnins.2018.01033 (PMC6336091; doi:10.3389/fnins.2018.01033)

Supplementary Figure 1. Experimental design.

Mice in ST groups were exposed to chronic unpredictable mild stress (CUMS) over a period of 8 weeks starting at the age of 14 weeks.

Supplementary Figure 2. Similar food and water consumption between WT and TG under both environmental conditions.

Food and water consumption in gram (g) was monitored in LabMaster system over a period of 22 hours. Data presented for individual mice with mean ± SEM for each group. Two-way ANOVA was performed.

Supplementary Figure 3. CUMS-induced expression changes in WT mice limited to few genes.

RNA sequencing results for four genes identified in WT_{ST}/WT_{SE} comparison (Fig. 5a). Shown are expression levels in normalized reads per kilobase per million (nRPKMs) as individual data points with mean ± SEM.

Supplementary Figure 4. Modest hippocampal gene expression changes upon CUMS exposure and *SNCA* overexpression.

(A) Number of differentially expressed genes for the main comparisons between the four experimental groups in hippocampus.
(B) Composition and expression level of hippocampal murine and human *SNCA* splice variants.
(C) Expression levels in normalized reads per kilobase per million (nRPKMs) as individual data points with mean ± SEM for *Slc17a6*.
(D) Expression levels in normalized reads per kilobase per million (nRPKMs) as individual data points with mean ± SEM for *Nr3c1* and *Nr3c2*.

Supplementary Figure 1

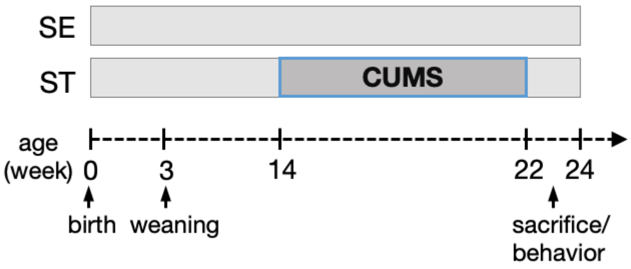

Supplementary Figure 2

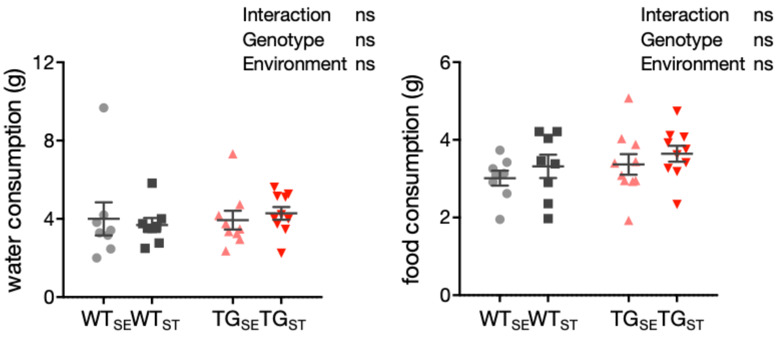

Supplementary Figure 3

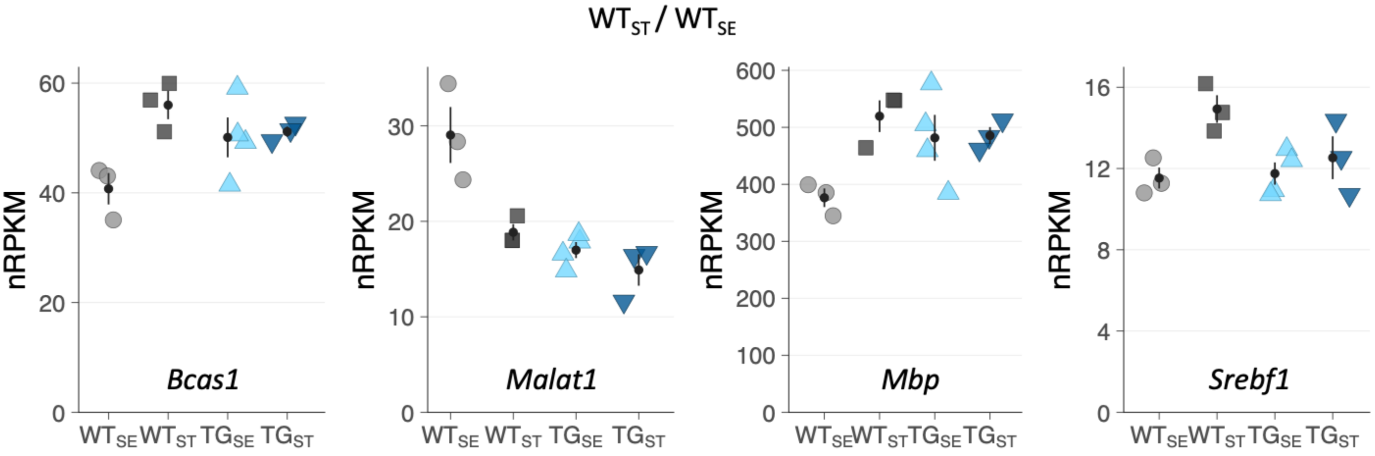

Supplementary Figure 4

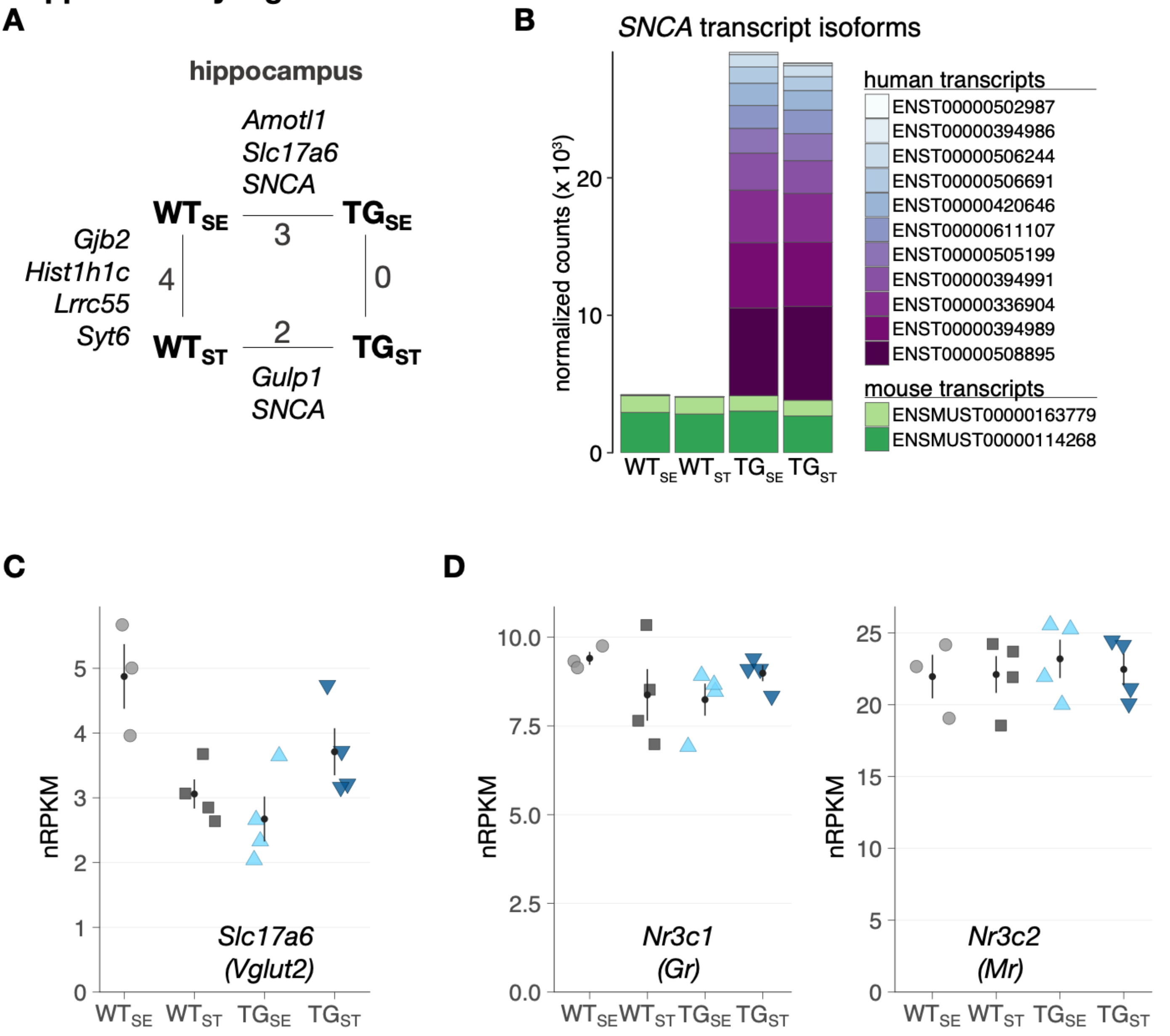

Supplement: Supplementary file 1 [file Data_Sheet_1.PDF]
